# Investigating the effect of paralogs on microarray gene-set analysis

**DOI:** 10.1186/1471-2105-12-29

**Published:** 2011-01-24

**Authors:** Andre J Faure, Cathal Seoighe, Nicola J Mulder

**Affiliations:** 1 Computational Biology Group, Department of Clinical Laboratory Sciences, University of Cape Town, Cape Town, South Africa; 2 EMBL-European Bioinformatics Institute (EBI), Wellcome Trust Genome Campus, Hinxton, Cambridge, UK; 3 School of Mathematics, Statistics and Applied Mathematics, National University of Ireland Galway, Ireland

## Abstract

**Background:**

In order to interpret the results obtained from a microarray experiment, researchers often shift focus from analysis of individual differentially expressed genes to analyses of sets of genes. These gene-set analysis (GSA) methods use previously accumulated biological knowledge to group genes into sets and then aim to rank these gene sets in a way that reflects their relative importance in the experimental situation in question. We suspect that the presence of paralogs affects the ability of GSA methods to accurately identify the most important sets of genes for subsequent research.

**Results:**

We show that paralogs, which typically have high sequence identity and similar molecular functions, also exhibit high correlation in their expression patterns. We investigate this correlation as a potential confounding factor common to current GSA methods using Indygene http://www.cbio.uct.ac.za/indygene, a web tool that reduces a supplied list of genes so that it includes no pairwise paralogy relationships above a specified sequence similarity threshold. We use the tool to reanalyse previously published microarray datasets and determine the potential utility of accounting for the presence of paralogs.

**Conclusions:**

The Indygene tool efficiently removes paralogy relationships from a given dataset and we found that such a reduction, performed prior to GSA, has the ability to generate significantly different results that often represent novel and plausible biological hypotheses. This was demonstrated for three different GSA approaches when applied to the reanalysis of previously published microarray datasets and suggests that the redundancy and non-independence of paralogs is an important consideration when dealing with GSA methodologies.

## Background

DNA microarray technology provides a high-throughput tool for gene expression analysis, and has revolutionised biological and biomedical research. The challenge of gaining biological insight from the inherently noisy raw expression data obtained from a microarray experiment has been met with numerous methodologies. Initially developed methods aim to identify individual genes whose expression levels differ or correlate significantly between two or more states, and typically result in a long list of genes for follow-up analysis or assay.

Subsequently, many methods proposed have shifted the focus from analysis of individual genes to sets of genes typically defined by their annotations to terms in databases such as the Gene Ontology (GO) [1], the Kyoto Encyclopaedia of Genes and Genomes (KEGG) [2] or the Molecular Signatures Database (MSigDB) [3]. These gene-set analysis (GSA) methods aim to rank these sets in a way that reflects their relative contributions to the observed gene expression changes in a particular experiment. The incorporation of an independent representation of previously accumulated biological knowledge into the analysis has proven to be powerful [4] and shifting the focus from individual genes to sets of genes has also been shown to identify biological themes more consistently across independent studies than results from single-gene analyses [3].

### GSA Methods and Tools

Using the classification system first defined by Pavladis *et al. *[5], GSA methods can be separated into two broad categories. The earliest and most popular methods use an overrepresentation analysis (ORA) approach, where the first step involves selecting a list of "interesting" genes based on their differential expression status, correlation with a phenotype or some other criteria. A contingency test is then performed to determine the functional terms or gene sets that are over- or underrepresented in this list when compared to the list of all genes considered in the experiment. There are currently over 20 tools that perform this type of analysis. Amongst these, a common choice of hypothesis test is Fisher's exact test, which is used by tools such as GoMiner [6], EASEonline [7] and FatiGO [8]. Other tools including CLENCH [9], GO::TermFinder [10] GOstat [11], GoSurfer [12] and the NetAffx GO Mining Tool [13] offer related tests based either on binomial, normal or *χ*^2 ^approximations to the hypergeometric distribution.

Methods using the ORA approach have been widely criticised, because they consider only the significant genes satisfying an arbitrary threshold and information about the continuous evidence supporting differential expression is lost [4]. The choice of threshold for determining "interesting" genes can severely influence the biological conclusions drawn from analyses using these methods [14]. More fundamentally, the statistical models themselves are considered inappropriate as they take the gene rather than the case as the sampling unit, relying on gene randomisation to assess significance. Implicit in these tests is also the highly unrealistic assumption that gene transcripts are expressed independently [15]. Despite these flaws, ORA methods are still used by researchers with surprising frequency.

Subsequently, a large number of methods have been developed to address the limitations of ORA and are collectively referred to as "functional class scoring" (FCS) methods. The methods in this second category differ from ORA in that they do not divide the data into two distinct groups of genes, but instead provide a score based on all genes within a particular class or sharing a common functional annotation. One of the most widely adopted FCS methods is Gene Set Enrichment Analysis (GSEA) [3,16], which starts by ranking the genes in a microarray experiment by their gene-level statistics. The ranked gene list is then used to calculate an enrichment score (*ES*) for each gene set, which reflects the tendency of genes in a particular set to occur towards the extremes of the list. Gene sets whose genes are non-uniformly distributed in the list are assigned a high *ES *and are expected to be more related to the gene expression differences observed. To assess the statistical significance of the *ES*, GSEA uses a sample randomisation approach.

GSEA has been shown to produce interesting and biologically relevant results [16], even in cases where no genes were found to be differentially expressed after a multiple testing correction was applied. Results from GSEA also indicate improvements in sensitivity and reproducibility over those obtained using ORA methods [3]. However a number of authors have criticised the approach for using a competitive strategy where the significance of a particular gene set can be affected by the presence or absence of other higher or lower ranking gene sets [4,17].

Other FCS methods have been proposed which improve upon GSEA and outperform this tool in terms of power [18]. More recently a general modular framework has emerged from the observation that all GSA strategies can be separated into a number of discreet steps enabling systematic comparisons [19]. The general scheme of the framework consists of a gene-level analysis, followed by the calculation of gene set statistics and finally significance assessment. The latter two steps are each motivated by a choice of null hypothesis - a factor of crucial importance to the overall GSA procedure. Exceptions to this structure include methods such as Global Test [20] and a related approach called ANCOVA Global Test [21], which model the entire gene set directly without the presence of separate intervening steps for the calculation of gene-level and gene set statistics (see [19] for a review).

Goeman and Buhlmann [15] first described the differences between various GSA methods in terms of their null hypotheses, where the two most common choices are termed the "competitive null hypothesis", $H_{0}^{comp}$ and the "self-contained null hypothesis", $H_{0}^{self}$. Their general formulations are given by,

H0comp : The genes in G are as often differentially expressedas the genes in Gc.

$$H_{0}^{self}\;:\text{ No genes in }G\text{ are differentially expressed}.$$

where *G *represents the gene set of interest and *G^c ^*its complement. ORA methods test a competitive null hypothesis and their use of gene randomisation to determine statistical significance follows naturally from this. On the other hand, assessing significance using sample randomisation is the intuitive alternative for methods testing a self-contained null hypothesis. These latter methods avoid issues relating to the competitive scoring of gene sets and the problematic assumptions made when performing gene randomisation to assess statistical significance. With these distinctions in mind, GSEA can be viewed as a hybrid GSA method in that its choice of a Kolmogorov-Smirnov-like statistic is motivated by a competitive null hypothesis, whereas it determines significance of each *ES *using sample randomisation. This is offered as a reason for its low power in some instances [15].

FCS methods with more appropriate choices of test statistic and significance assessment include the global procedures mentioned above as well as SAM-GS [18], and all make use of a self-contained null hypothesis. The Global Test tests whether subjects (or microarray experiments) with similar gene expression profiles have similar class labels, using a logistic regression model. Applied to a gene set, it tests how well the expression profiles of the member genes are able to predict the class labels. The Global Test is versatile in that it can be applied in diverse microarray experimental design situations including two-class, multi-class, continuous and survival outcome types [22]. Compared to Global Test, ANCOVA Global Test has the roles of class labels and gene expression profiles exchanged in the regression model, and its authors point out that it performs better than Global Test when strong dependencies exist between genes.

The standard SAM procedure [23] uses a t-like statistic to test whether an individual gene in a microarray experiment is differentially expressed and was developed in this context to stabilise small variances. SAM-GS extends the SAM procedure to identify gene sets showing significant differential expression. The null hypothesis is self-contained and states that a gene set is not differentially expressed across a two-class phenotype. SAM-GS evaluates significance by way of sample randomisation, where a *P*-value is calculated by comparing the test statistic to its null distribution obtained by permuting the microarray class labels many times. Importantly, SAM-GS was developed to detect bidirectional gene expression changes. Therefore, a significant *P*-value merely indicates that the genes in the gene set exhibit substantial expression change between the two phenotype classes without distinguishing between differentially up- or down-regulated genes. Liu *et al. *[24] performed a comparative evaluation of the three aforementioned GSA methods using a simulation experiment and three real-world microarray datasets. All three methods display similar performance, except SAM-GS exhibits slightly higher power with regard to highly significant gene sets.

Other methods that fall into the category of FCS include PathwayRF [25] and the Learner of Functional Enrichment (LeFE) [26], which both use machine learning approaches to analyse gene expression data in terms of gene sets. Both of these methods model the expression data as an ensemble of decision trees (Random Forest), an advantage of which is that they have the potential to capture complex nonlinear relationships that may exist between genes in a gene set. A number of methods have also taken a systems biology approach to GSA by incorporating pathway topology information. Examples include ScorePAGE [27] and Pathway-Express [28].

### Motivation for Indygene

There has been a flood of GSA tools developed in recent years, but to our knowledge no research directed at evaluating the effect of the presence of paralogs on results from these analyses. Typically paralogy is inferred on the basis of sequence similarity between genes, or sequence or structural similarity between the proteins they encode. Additionally it is well known that paralogs show a high degree of functional similarity - large-scale automatic annotations of gene products to functional terms in databases such as the GO have long exploited this fact. We show that paralogs also display high correlation in their expression patterns. This suggests that paralogs exhibit three-fold redundancy i.e. in sequence, expression and function and has led us to suspect that paralogs may influence results from GSA in an undesired manner as these methods involve comparisons between two of these factors. A clear example of this follows from the observation that the expression correlation of paralogs is at odds with the assumption of gene expression independence made by GSA methods such as ORA, which use gene resampling to assess significance.

Gene duplication seems to be a general mechanism of adaptation to various environmental stresses, in both prokaryotic and eukaryotic species, and paralogs can become fixed in a population, at least initially, as a result of their effect on gene dosage [29]. In such cases, the number of genes associated with a particular cellular process is more a response to environmental conditions or stoichiometric requirements than a reflection of the level of coordination or complexity required within the associated pathway or cellular process. However GSA methods in general regard all genes that are members of a particular gene set or annotated to a particular functional term as equally important in determining the importance of that gene set. Therefore results from apparently statistically sound procedures, such as SAM-GS, that avoid problematic assumptions of expression independence are still likely to be biased by the presence of paralogs in that this represents the inclusion of, at worst, essentially redundant information.

The ScorePAGE algorithm [27], which scores changes in the activity of metabolic pathways using microarray data, seems to represent an interesting exception to this general rule. To avoid the inclusion of redundant information, the authors abstract the analysis away from the level of genes to enzymes, which are the most relevant entities, by selecting one representative gene when their products catalyze the same enzymatic reaction. Similarly, we argue that it is desirable to account for biases represented by the presence of redundant information in the form of paralogs. To highlight these issues and assess the extent of the problem, we developed the Ingygene tool, which efficiently removes paralogy relationships from a given dataset. Although this does not eliminate the bias resulting from the presence of paralogs, it is expected to at least alleviate the problems relating to redundancy and non-independence of their expression patterns. We applied Indygene to the reanalysis of previously published microarray datasets and our results suggest that such a reduction, performed prior to GSA, has the ability to generate significantly different results often representing novel and plausible biological hypotheses. This was demonstrated for GoMiner, GSEA and SAM-GS, which together cover a substantial portion of the GSA taxonomy, both from the point of view of the classification of the null hypothesis, as well as the distinction made between ORA and FCS.

## Results and Discussion

### Coexpression of Paralogs

We used gene and protein sequence data together with a large collection of gene expression experiments to determine the extent to which paralogs have correlated expression patterns using *Arabidopsis thaliana *as a case study. We determined candidate paralogs in *Arabidopsis *using its entire proteome and a two-step procedure involving the all-against-all comparison of 35007 *Arabidopsis *protein sequences from UniProtKB and the global alignment and scoring of 982254 pairs with BLAST E-values below a cut-off threshold of 10^-5^. Rost [30] found that 90% of protein pairs with greater than 30% sequence identity were homologous, whereas below 25% identity less than 10% were and below 20% identity there is an explosion in the number of false positives. We therefore proceed by considering only those protein pairs with sequence identity > 20%.

**Figure 1:**
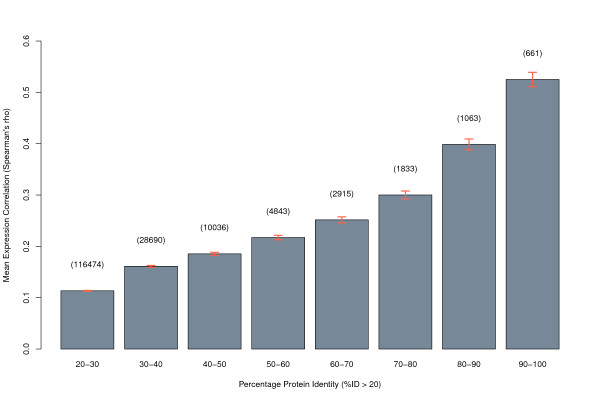
**Paralog expression correlation**. Mean expression correlation (Spearman's *ρ*) of gene paralogs in *Arabidopsis *at various protein sequence identity levels where %ID > 20. Error bars indicate the standard error of the estimated mean values. The values in parentheses indicate the number of unique pairwise gene comparisons in each case.

After attributing gene names to all protein pairs and removing redundant gene matches and those without associated gene name information, 677473 gene pairs remained. We investigated the coexpression of paralogs using a large *Arabidopsis *microarray gene expression dataset from the Nottingham Arabidopsis Stock Centre's (NASC) AffyWatch service [31]. Gene expression values across multiple experiments were used to calculate correlation values for each paralog pair. We were able to estimate Spearman's rank correlation coefficient for 409944 gene pairs (not all paralogs found were represented on the microarray platform used) and Figure 1 shows the mean expression correlation for paralogs at different levels of protein identity. Gene expression correlation of paralogs showed a clear tendency to increase with increasing protein sequence similarity. On average, paralogs with 90-100% protein sequence identity have a strong correlation ($\bar{\rho }>0.5$) in their gene expression patterns.

As these expression correlation values are based on over 1500 individual microarray experiments, the results presented here are highly statistically significant and provide evidence in support of the notion that the regulatory and coding sequences of paralogs tend to co-diverge [32]. The extent of the observed correlation in the expression patterns of paralogs also warrants further investigation in terms of their effect on results from microarray GSA.

### Indygene Tool

**Figure 2:**
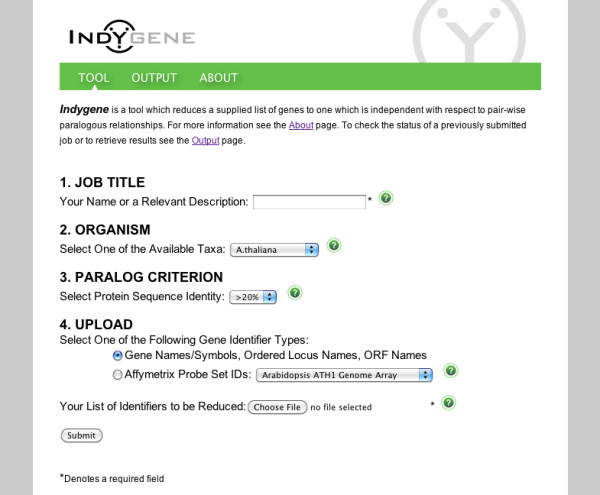
**Indygene 'Tool' page**. Indygene 'Tool' page showing the form used to submit a gene list for processing.

The Indygene tool http://www.cbio.uct.ac.za/indygene reduces a supplied list of genes to one without paralogy relationships, where the goal is to proceed with GSA thereafter. The web tool consists of a simple interface allowing the user to submit a list of gene or Affymetrix probe identifiers and select a protein sequence identity threshold for paralogy (see Figure 2). Currently the following taxa are available for selection: *Arabidopsis*, human, mouse and rat. In addition, the following Affymetrix microarray platforms are available: Arabidopsis ATH1 Genome Array, Arabidopsis Genome Array, Human Genome Focus Array, Human Genome U133 Array Plate Set, Human Genome U133 Plus 2.0 Array, Human Genome U133 Set, Human Genome U133A 2.0 Array, Human Genome U95 Set. The 'Output' page provides links to the reduced gene list and log file. The former contains gene identifiers in their originally submitted format ready for use in conjunction with any preferred GSA tool.

The Indygene algorithm calculates paralogy relationships between the genes in the user-submitted gene list, which can be represented in a graph *G *as edges and vertices respectively. Reducing a list of genes to one without paralogy relationships is equivalent to finding a stable set in *G*, which is a set of vertices (genes) that are mutually nonadjacent i.e. no two vertices are connected by an edge (paralogy relationship). Typically such graphs contain many stable sets of different sizes, but in view of the cost and time involved in generating gene expression data, we are interested in obtaining the largest stable set possible so as to retain the maximum amount of information for further analysis. This is the optimisation version of the stable set problem, called the maximum stable set problem (MSSP), which attempts to find the largest stable set in *G*. MSSP is known to be an NP-complete problem [33] and therefore there are no efficient algorithms to calculate its exact solution in a reasonable amount of time.

**Figure 3:**
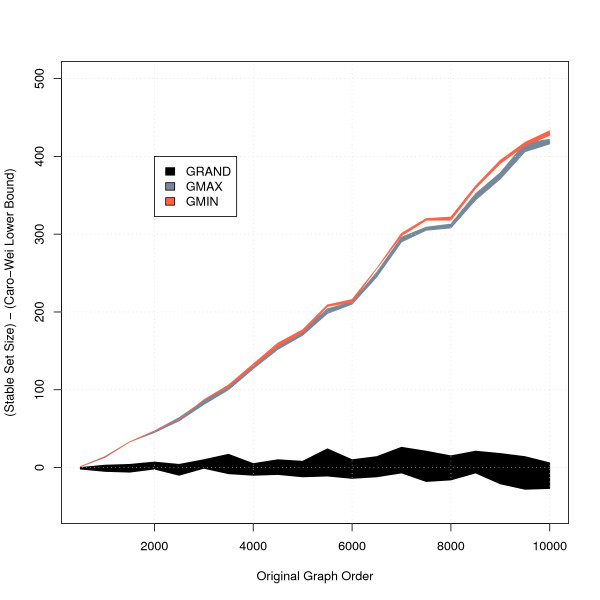
**Comparison of stable set sizes obtained using three different algorithms for the MSSP**. Graph order before and after the application of three greedy algorithms for the MSSP to random *Arabidopsis *gene graphs of differing sizes. We indicate the stable set size range over 10 replicates in each case. The ordinate shows the number of genes by which the stable set size exceeds the lower bound given by the Caro-Wei theorem.

**Figure 4:**
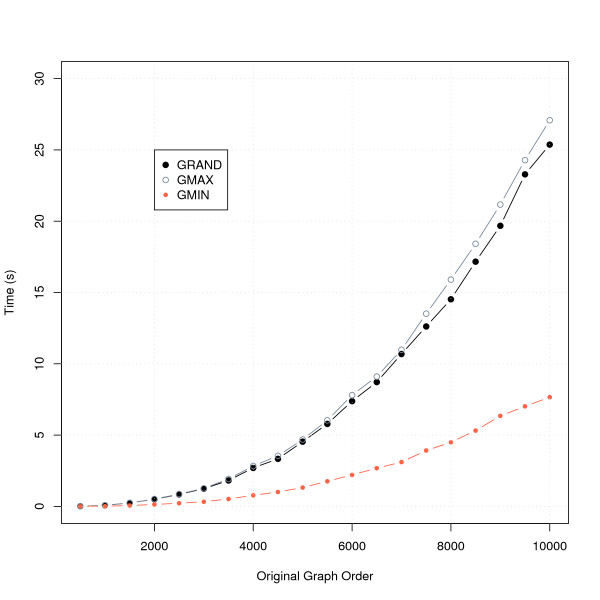
**Comparison of computation times of three different algorithms for the MSSP**. Mean computation times of three greedy algorithms for the MISP applied to random *Arabidopsis *gene graphs of differing orders. We indicate the mean calculation time over 10 replicates in each case.

We considered three greedy algorithms that provide approximate solutions to the MSSP, namely GRAND, GMAX and GMIN. If *α*(*G*) is the size of the maximum stable set in *G *and *d*(*v*) is the degree of vertex *v*, Caro [34] and Wei [35] both independently showed that

$$\alpha (G)\geq \sum_{v\in V}1/[d(v)+1]$$

which has subsequently been referred to as the Caro-Wei theorem [36]. Furthermore, for a graph *G *with degree bounded by Δ, Halldorsson and Radhakrishnan [37] proved that GMIN guarantees a lower bound on stable set size of at least 3*α*(*G*)/( Δ + 2), which is greater than that of GMAX. The results of the practical performance of the three algorithms when applied to gene graphs created using randomly generated lists ranging in length from 500 to 10000 randomly selected *Arabidopsis *genes are shown in Figure 3 and Figure 4. Both GMIN and GMAX improve on solutions from GRAND by hundreds of genes when the graph order is high, with GMIN finding solutions at least as large as those found by GMAX. In terms of computational time, Figure 4 shows that GMIN is the most time-efficient algorithm. We therefore adopted an optimised version of this algorithm in the Indygene tool.

### Removing paralogs leads to significantly different GSA results

If the presence of paralogs does indeed adversely affect results from GSA, the elimination of paralagous relationships in a gene expression dataset should lead to significantly different GSA results. To investigate this we used the dataset of Alonso *et al. *[38] who measured genome-wide expression changes in plants in response to ethylene. We used Indygene to determine whether performing a paralog-reduction on this dataset prior to GSA tends to generate results that are significantly different from those obtained after random reductions of the same number of genes.

**Figure 5:**
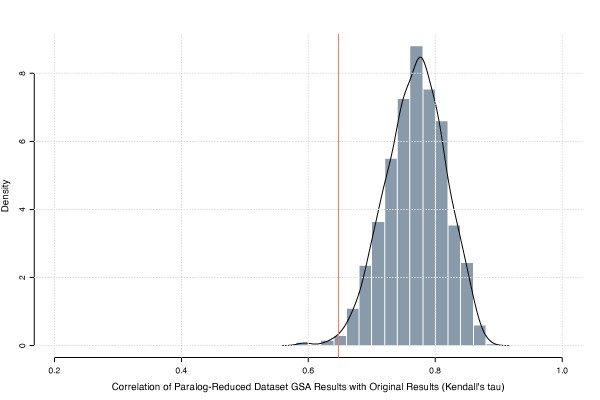
**Removing paralogs leads to significantly different GSA results**. Estimated null distribution for *τ *used to determine whether the paralog-reduced dataset (red vertical line at *τ *= 0.65) produces significantly different GSA results. The abscissa gives Kendall's correlation (*τ*) between the ranked GO term lists before and after randomly reducing the dataset by 6126 genes. The black line indicates the approximate probability density function of the null distribution, estimated using a Gaussian smoothing kernel.

We performed GSA on the original microarray dataset using an ORA approach (see Methods) and compared the resulting list of GO SLIM terms from the Biological Process ontology to that obtained after reducing the dataset by 6126 genes using Indygene. The obtained correlation value of *τ *= 0.65 quantifies the difference between the ranking of terms in the two lists. To determine whether this difference was statistically significant and not merely related to the removal of a large number of genes, we estimated the null distribution for *τ *using a Monte Carlo sampling procedure (see Figure 5). When compared to this null distribution, a nonparametric *P*-value ≈ 0:007 was obtained, indicating that the presence of paralogs can significantly affect results from GSA. In other words, a paralog reduction as performed by Indygene can result in a significantly different GSA term ranking, not simply attributable to the reduction in the number of genes.

### Reanalysis of Previously Published Datasets

Having determined that removing paralogs significantly alters the relative ranking of gene set results, we now ask whether these novel results can represent plausible hypotheses regarding the biological processes underlying the response under study, in a way that is particular to the paralog-reduced dataset. To this end we reanalysed previously published gene expression datasets using three tools which make use of diverse statistical methodologies: GoMiner, GSEA and SAM-GS. The rationale was to establish whether performing GSA on paralog-reduced datasets could reveal novel and biologically relevant themes not otherwise identified. However with respect to these comparisons, it should also be noted that any discrepancies highlighted between different sets of results are anecdotal and not intended to show definitive benefits or drawbacks of either approach.

### Reanalysis of GoMiner Results Using Indygene

There are numerous tools that use the ORA approach, however GoMiner was amongst the first to be developed and has proven to be one of the most popular tools with almost 700 academic citations to date [39]. Spira *et al. *[40] used GoMiner to find terms in the GO Molecular Function ontology that were associated with genes expressed in the airway epithelial cells of healthy never-smokers, and therefore also associated with these cells' normal functioning. We repeated the analysis using the original and paralog-reduced gene lists and terms from the GO Biological Process ontology. Using the same *P*-value cut-off of *α *= 0.05 used by Spira *et al. *[40], 118 GO terms were found to be significant both before and after reduction, but there were also additional terms found to be significant in each case. Table 1 shows only those terms exclusive to the results obtained from either the original or paralog-reduced list.

**Table 1:** GoMiner GSA results

Original dataset: unique GSA results	Reduced dataset: unique GSA results
GO:0006007 - glucose catabolic process	GO:0009225 - nucleotide-sugar metabolic process
GO:0009056 - catabolic process	GO:0042632 - cholesterol homeostasis
GO:0002504 - antigen processing and presentation of peptide...	GO:0006890 - retrograde vesicle-mediated transport Golgi to ER
GO:0019320 - hexose catabolic process	GO:0000059 - protein import into nucleus docking
GO:0046365 - monosaccharide catabolic process	GO:0006692 - prostanoid metabolic process
GO:0006096 - glycolysis	GO:0006693 - prostaglandin metabolic process
GO:0012501 - programmed cell death	GO:0006183 - GTP biosynthetic process
GO:0019882 - antigen processing and presentation	GO:0007368 - determination of left right symmetry
GO:0006915 - apoptosis	GO:0008543 - fibroblast growth factor receptor signaling pathway
GO:0051258 - protein polymerization	GO:0009799 - determination of symmetry
GO:0008219 - cell death	GO:0009855 - determination of bilateral symmetry
GO:0016265 - death	GO:0030520 - estrogen receptor signaling pathway
GO:0031529 - ruffle organization and biogenesis	GO:0046039 - GTP metabolic process
GO:0048259 - regulation of receptor mediated endocytosis	
GO:0045045 - secretory pathway	
GO:0044275 - cellular carbohydrate catabolic process	
GO:0006006 - glucose metabolic process	
GO:0048193 - Golgi vesicle transport	
GO:0030832 - regulation of actin filament length	
GO:0007018 - microtubule-based movement	
GO:0016052 - carbohydrate catabolic process	
GO:0001508 - regulation of action potential	
GO:0043067 - regulation of programmed cell death	
GO:0006879 - iron ion homeostasis	
GO:0042981 - regulation of apoptosis	
GO:0007265 - Ras protein signal transduction	
GO:0030032 - lamellipodium biogenesis	
GO:0009894 - regulation of catabolic process	
GO:0032940 - secretion by cell	
GO:0007010 - cytoskeleton organization and biogenesis	
GO:0040017 - positive regulation of locomotion	
GO:0051272 - positive regulation of cell motility	
GO:0006402 - mRNA catabolic process	
GO:0006471 - protein amino acid ADP-ribosylation	
GO:0008064 - regulation of actin polymerization ...	
GO:0030036 - actin cytoskeleton organization ...	
GO:0048468 - cell development	
GO:0005996 - monosaccharide metabolic process	
GO:0006996 - organelle organization and biogenesis	

GoMiner GSA results indicating GO Biological Process terms significantly overrepresented amongst the genes expressed in airway epithelial cells from never-smokers. Only those terms exclusive to the results obtained from either the original or paralog-reduced list are shown. A *P*-value cut-off of *α *= 0.05 was used to determine significance.

A number of interesting GO biological process terms were only found to be significant when GoMiner was applied to the paralog-reduced dataset. Their role in the normal functioning of airway epithelial cells seems plausible given the supporting evidence in the literature. For instance Babiker *et al. *[41] found that the human lung plays an important role in maintaining 'cholesterol homeostasis' (GO:0042632) by the elimination of cholesterol as cholestenoic acid. Also, it is well known that smoking is associated with increased HDL cholesterol levels [42], possibly explained by the malfunctioning of this homeostatic process caused by exposure to tobacco smoke.

Prostaglandins are a major product of airway epithelium and different types are involved in functions such as bronchodilation and bronchoconstriction. Endogenous Prostaglandin E2 (PGE2) has been found to control the latter [43]. It is therefore not surprising that 'prostaglandin metabolic process' (GO:0006693) and its parent term 'prostanoid metabolic process' (GO:0006692) were found to be overrepresented amongst the expressed genes. Also, Mollerup *et al. *[44] found that estrogen receptors are expressed in normal lung tissue in both sexes, enabling the 'estrogen receptor signalling pathway' (GO:0030520) to respond to the hormone, which is required for the promotion of lung function by the maintenance of alveoli [45].

Two of the other remaining terms (GO:0009799 'determination of symmetry', GO:0009855 'determination of bilateral symmetry') also seem to represent plausible biological processes given the importance of airway symmetry. These results show that performing GSA on a paralog-reduced expression dataset using the ORA approach can yield novel and biologically relevant terms not otherwise identified.

### Reanalysis of GSEA Results Using Indygene

GSEA is an extremely popular tool amongst biologists and, despite widespread criticism, has been recommended for the analysis of human gene expression data in recent reviews of GSA methods [4,46]. We compared the differences between the results from the reanalysis of five different GSEA gene expression datasets before and after eliminating paralogy relationships using Indygene. The five datasets cover a diverse range of topics, namely gender-specific expression differences in lymphoblastoid cells, p53 status in cancer cell lines, classification of acute leukaemias and two lung cancer outcome studies. Subramanian *et al. *[3] used these datasets to show GSEA's ability to detect subtle but coordinated expression changes in sets of related genes defined by MSigDB. MSigDB consists of five major collections of human gene sets, but we only make use of two of these: MSigDB:C1, which contains gene sets based on chromosomal location and MSigDB:C2, which contains gene sets based on common roles in metabolic/signalling pathways or coregulation in response to chemical/genetic perturbations. The results of our analyses, which were obtained using the same significance threshold as these authors, are shown in Table 2.

**Table 2:** GSEA GSA results

Original dataset: unique GSA results	Reduced dataset: unique GSA results
[1] Lymphoblast cell lines:	[1] Lymphoblast cell lines:
**- Enriched in males:**	**-Enriched in males:**
TGFBETA_C2_UP	CROONQUIST_IL6_STROMA_UP
**-Enriched in females:**	**-Enriched in females:**
	CHESLER_HIGHEST_FOLD_RANGE_GENES BHATTACHARYA_ESC_UP

**[2] p53 status in NCI-60 cell lines:**	**[2] p53 status in NCI-60 cell lines:**
**-Enriched in p53 wild type:**	**-Enriched in p53 wild type:**
P53HYPOXIAPATHWAY	
HSP27PATHWAY	
MMS_HUMAN_LYMPH_HIGH_24HRS_UP	
P53PATHWAY	
KANNAN_P53_UP	
P53_BRCA_UP	
RADIATION_SENSITIVITY	

**[3] Acute leukaemias:**	**[3] Acute leukaemias:**
**-Enriched in ALL:**	**-Enriched in ALL:**
	chr13q14

**[4] Lung cancer outcome (Boston study):**	**[4] Lung cancer outcome (Boston study):**
**-Enriched in poor outcome:**	**-Enriched in poor outcome:**
TGFBETA_C1_UP HDACLCOLON_TSA_DN	CANCER_UNDIFFERENTIATED_META_UP
	MARSHALL_SPLEEN_BAL
	TRNASYNTHETASES
	EGF_HDMEC_UP
	AMINOACYL-TRNA_BIOSYNTHESIS
	ZELLER_MYC_UP
	HDACI_COLON_BUT16HRS_DN
	ZHAN_MULTIPLE_MYELOMA_SUBCLASSES_DIFF
	MYC_TARGETS
	MENSE_HYPOXIA_UP
	SMITH_HTERT_UP
	DOX_RESIST_GASTRIC_UP
	BASSO_REGULATORY_HUBS

**[5] Lung cancer outcome (Michigan study):**	**[5] Lung cancer outcome (Michigan study):**
**-Enriched in poor outcome:**	**-Enriched in poor outcome:**
TGFBETA_C1_UP	
HSA00010_GLYCOLYSIS_AND_GLUCONEOGENESIS	
GLYCOLYSIS GLUCONEOGENESIS	
MENSE_HYPOXIA_UP	
VEGFPATHWAY	
ROME_INSULIN_2F_UP	
INSULIN-SIGNALING	
BHATTACHARYA_ESC_UP	
VANTVEER_BREAST_OUTCOME_GOOD_VS_POOR_DN	
GLYCOLYSIS_AND_GLUCONEOGENESIS	
ZUCCHI_EPITHELIAL_DN	
HYPOXIA_REVIEW	

GSEA results of five diverse gene expression datasets showing gene sets significantly enriched in the phenotype indicated. Functional gene sets (MSigDB:C2) were used in all cases, except for the leukaemia dataset where cytogenetic gene sets (MSigDB:C1) were used. Only those sets exclusive to the results obtained from either the original or paralog-reduced list are shown. A threshold of FDR ≤ 0.25 was used to determine significance.

The original and paralog-reduced results for the lymphoblast cell lines dataset were similar, with the latter revealing one gene set enriched in males and two in females, which did not occur with the former. According to MSigDB:C2, the gene set attributed to work by Croonquist *et al. *[47] indicates "genes upregulated in multiple myeloma cells exposed to the pro-proliferative cytokine IL-6 versus those co-cultured with bone marrow stromal cells". The relevance of this gene set and the sets based on studies by Chesler *et al. *[48] and Bhattacharya *et al. *[49] to gender-specific expression differences is not clear. The significance of these gene sets may be artifactual and due to confounding factors such as a gender-biased sampling programme in these studies.

No significantly enriched gene sets unique to the paralog-reduced 'p53 status' dataset were found i.e. the one gene set identified is shared with the original results. However, the one significantly enriched gene set unique to the paralog-reduced 'acute leukaemias' dataset corresponds to the 13q14 cytogenetic location ('MSigDB:C1:chr13q14') containing the *RB *gene, which is often deleted or translocated in patients with AML, but rarely in ALL [50]. This evidence confirms the importance of this gene set regarding expression differences between acute leukaemia subclasses.

For the 'Michigan lung cancer outcome' study, the paralog-reduced results include three significant gene sets shared with the original results, but no unique sets. The Boston counterpart, on the other hand, yielded many significantly enriched gene sets unique to the paralog-reduced dataset and a number of these are plausible contributors to the poor outcome observed. 'MSigDB:C2:CANCER_UNDIFFERENTIATED_META_UP' is a gene set comprised of 69 genes commonly upregulated in undifferentiated cancer. Undifferentiated cancers tend to be more malignant than well-differentiated cancers, possibly explaining the association between this gene set and a poor survival outcome. Also, the 'MSigDB:C2:ZELLER_MYC_UP' and 'MSigDB:C2:MYC_TARGETS' gene sets contain genes that are up-regulated, or otherwise responsive, to *Myc*. The *Myc *protein is a transcription factor that stimulates the expression of many genes involved in cell-cycle progression. Its overexpression has also been associated with many types of cancer [51]. Furthermore, Berns *et al. *[52] and Grotzer *et al. *[53] found that high *Myc *expression is correlated with a poor outcome in patients with breast and brain cancers respectively. It is conceivable that a similar relationship exists in the case of lung cancer. Explanations for the other significantly enriched gene sets unique to the paralog-reduced dataset are not apparent, but could provide novel hypotheses for future investigation.

### Reanalysis of SAM-GS Results Using Indygene

To benchmark the performance of SAM-GS against GSEA, Dinu *et al. *[18] reanalysed the 'p53 status' dataset of Subramanian *et al. *[3] discussed above. They used SAM-GS to analyse this dataset and identify MSigDB gene sets exhibiting bi-directional expression change across a two-class phenotype, defined by the presence or absence of the wild-type *p53 *gene. The results of our SAM-GS analysis using the original and paralog-reduced datasets are shown in Table 3. In addition to the unique gene sets in this table, there were 43 gene sets shared between the two sets of results. Here we focus on the most important gene sets satisfying the stricter significance threshold of FDR ≤ 0.001, as the original authors' threshold of FDR ≤ 0.01 resulted in about one hundred significant terms in each case.

**Table 3:** SAM-GS GSA results

Original dataset: unique GSA results	Reduced dataset: unique GSA results
APOPTOSIS	ADIP_VS_FIBRO_DN
APOPTOSIS-GENMAPP	BCNU_GLIOMA_MGMT_48HRS_UP
APOPTOSIS_KEGG	BRCA1_SW480_DN
CELLCYCLEPATHWAY	BREAST_CANCER_ESTROGEN_SIGNALING
CHEMICALPATHWAY	DAC_PANC50_UP
FSH_HUMAN_GRANULOSA_UP	DNA_DAMAGE_SIGNALING
G1PATHWAY	DRUG_RESISTANCE_AND_METABOLISM
HSA05219_BLADDER_CANCER	G2PATHWAY
HSP27PATHWAY	HSA05040_HUNTINGTONS_DISEASE
IL4PATHWAY	OXSTRES_BREASTCA_UP
P53_BRCA1_UP	PARP_KO_UP
RACCYCDPATHWAY	PASSERINI_APOPTOSIS
SA_FAS_SIGNALING	SA_DIACYLGLYCEROL_SIGNALING SHEPARD_NEG_REG_OF_CELL_PROLIFERATION

SAM-GS results indicating functional gene sets (MSigDB:C2) significantly enriched in the expression patterns of NCI-60 cancer cell lines with wild-type *p53*, compared to those of *p53 *mutants. Only those terms exclusive to the results obtained from either the original or paralog-reduced list are shown. A threshold of FDR ≤ 0.001 was used to determine significance.

The transcription factor p53 plays an important role in the cellular response to DNA damage. Because cells in cancerous tissue possess mutations in their DNA, up-regulation of *p53 *and other downstream 'MSigDB:C2:DNA_DAMAGE_SIGNALING' genes is to be expected for such cells with the wild-type. The wild-type *p53 *protein also has the ability to arrest cells with damaged DNA at particular points in the cell-cycle to avoid copying of these errors and provide time for their repair [51]. This anti-proliferative effect of wild-type p53 is evident in the significance of the 'MSigDB:C2:SHEPARD_NEG_REG_OF_CELL_PROLIFERATION' gene set, described as containing human genes whose orthologs in zebra fish negatively regulate cell proliferation. Another interesting gene set unique to the paralog-reduced results is 'MSigDB:C2:DRUG_RESISTANCE_AND_METABOLISM'. The findings of Bunz *et al. *[54], which seem to corroborate this result, show that the presence of mutations affecting *p53 *in human cancer cells renders them resistant to certain drugs used in cancer therapy. These results show that despite the statistical validity of the procedure used in SAM-GS, removing paralogy relationships in the data does influence the results, highlighting novel and biologically plausible hypotheses in the form of significantly enriched gene sets not found in the original dataset. Although we have only performed this analysis for SAM-GS, these results suggest that the presence of paralogs is likely to affect similar FCS methods, potentially obscuring the importance of relevant and promising gene sets for follow-up analysis.

## Conclusions

GSA often represents the first attempt to make biological sense of the data obtained from a microarray, or in fact any high-throughput experiment, and these methods enable the generation of hypotheses regarding the experiment. Promising hypotheses are usually investigated by means of further experimentation and therefore the accuracy of results from GSA has a direct impact on the amount of time, effort, money and success associated with future studies. We investigated the effect of paralogs on the results from GSA as we suspect that their non-independent expression patterns and, at worst, redundant molecular functions represents an unwanted bias.

As expected, we found that paralogs tend to have correlated expression patterns and their removal significantly affects results from GSA. The Indygene tool we developed efficiently removes paralogy relationships from a given dataset and we found that such a reduction, performed prior to GSA, has the ability to generate novel and biologically plausible hypotheses not otherwise obtained. Often the number of gene sets identified after removing paralogs is greater than that obtained when using the original data, suggesting that this reduction does not simply result in a reduction in the number of significant gene sets found.

We do not consider Indygene to be a permanent solution to problems caused by paralogous. In the absence of more sophisticated approaches, it should rather be regarded as a tool, that can be used alongside any GSA method, to explore the potential impact of the presence of paralogs on the results obtained. Future GSA methods should behave in such a way that the weight of evidence implicating a particular biological process based on the coordinated expression change of the participating members is greater when they have dissimilar sequences and distinct molecular functions. This may warrant a Bayesian statistical approach to GSA where the prior probability that paralogs exhibit coordinated expression change is greater than that of unrelated genes. Similarly, GSA methods taking a systems biological approach should account for the uniqueness of each gene's functional role and relative contribution in its associated biological pathway. Aggregating the expression response of groups of related genes with similar functions, before performing GSA on these "metagenes", could be another possible approach.

## Methods

### Paralog Prediction

*Arabidopsis thaliana *protein sequences were retrieved from the UniProt Knowlegebase (UniProtKB) [55], and candidate paralogs were identified by running an all-against-all BLASTP **(**blastall search, using an expectation value (*E*-value) cut-off of 10^-5 ^and a BLOSUM62 amino acid substitution matrix. We subsequently obtained a global percentage sequence identity measure (%ID) for candidate paralogs by performing a global alignment using an implementation of the Needleman-Wunsch algorithm (needle program) from the EMBOSS [56] software suite.

### Calculation of Expression Correlation

We used information from UniProt entries to assign gene names to each *Arabidopsis thaliana *protein pair and removed duplicate and self-matching gene entries (where multiple isoforms are encoded by a single gene) from the list of candidate paralogs. We then used Affymetrix GeneChip (microarray) data from the Nottingham Arabidopsis Stock Centre's (NASC) AffyWatch service [31] to determine whether gene paralogs exhibit correlation in their expression patterns. The data consists of gene expression measurements from over 1500 ATH1 GeneChips used in diverse experiments. After removal of outlier arrays, multiple array normalisation was carried out using the GCRMA (GC robust multi-array average) method [57]. We calculated expression correlation values for all pairs of genes in the list using this normalised meta-dataset. When more than one Affymetrix probe set identifier (probeID) was available for a particular gene, we attempted to select the most reliable one based on probeID suffix descriptions. To quantify gene expression correlation, we used Spearman's rank correlation coefficient (Spearman's *ρ*). For the calculations we used a custom script and the RPy package [58] to enable use of the necessary statistical functions in the R Programming Language [59].

### Comparison of Greedy Algorithms for the MSSP

**Figure 6:**
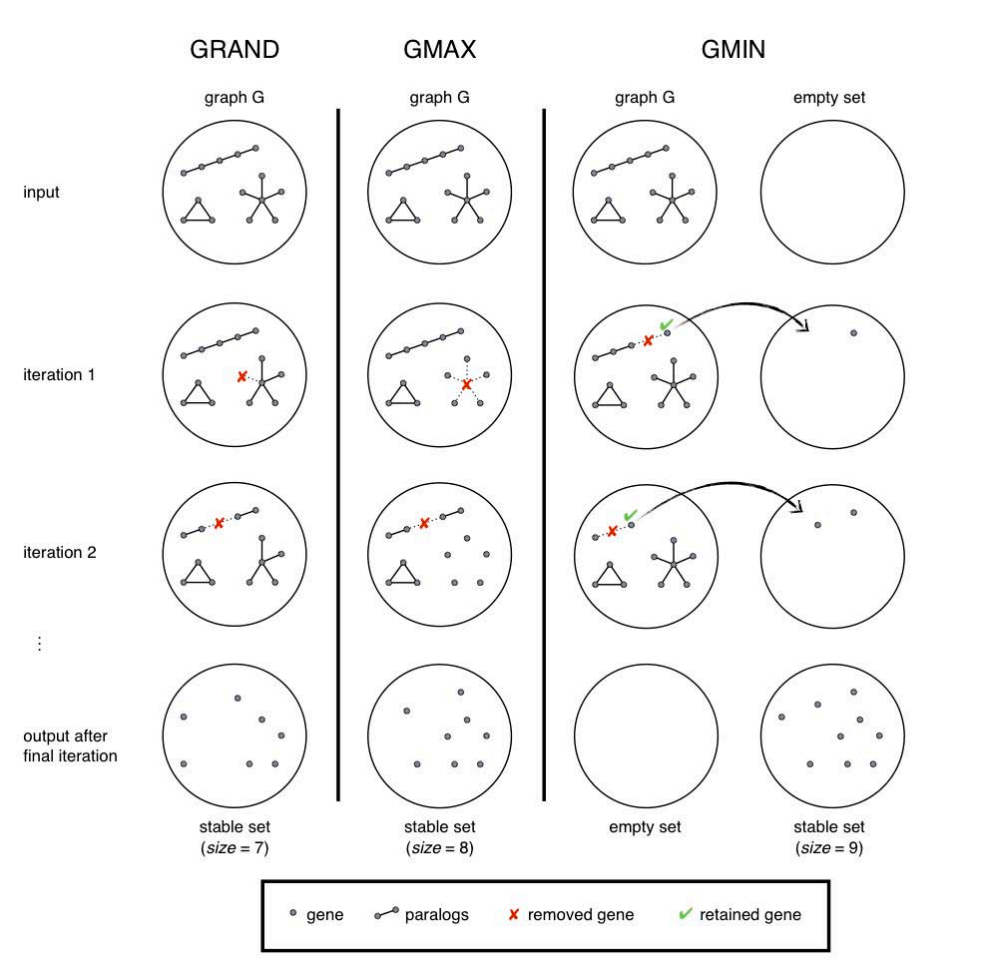
**Comparison of three greedy algorithms for the MSSP using a toy example**. A simulated graph *G *representing the paralogy relationships between 14 genes serves as input to each algorithm considered: GRAND, GMAX, GMIN. The final stable set of genes as well as the resulting graphs after two initial iterations are shown. Each iteration of GRAND consists of the removal of a random vertex (gene) whereas GMAX removes a vertex of maximum degree. This is repeated until no edges remain and the resulting set of genes is stable. GMIN selects a vertex of minimum degree to retain during each iteration and all adjacent vertices are removed. The process is repeated until *G *becomes empty and the retained vertices form a stable set.

Consider a graph *G *representing a list of *m *genes and the paralogy relationships between them as vertices and edges respectively. A number of graph theoretic algorithms can be used to find approximate solutions to the maximum stable set problem (MSSP) applied to *G*. We evaluated three such algorithms: GRAND, GMAX and GMIN, all of which use a greedy strategy. The simplest algorithm, GRAND, randomly removes vertices with non-zero degree until the resulting sub-graph is stable. GMAX is similar to GRAND, however instead of randomly removing vertices, a vertex of maximum degree is removed at each step. GMIN differs from the preceding two algorithms in that it selects a vertex of minimum degree to retain at each step. The selected vertex and all of its adjacent vertices are then removed from the remaining graph. The process is repeated until *G *becomes empty and the retained vertices form a stable set. See Figure 6 for a comparison of the GRAND, GMAX and GMIN algorithms using toy examples.

To evaluate the performance of these algorithms we implemented them using custom Python scripts and applied them to real-world data relevant to the intended application. The data comprised lists ranging in length from 500 to 10000 randomly selected Arabidopsis genes and we indicated paralogy relationships between gene pairs if their pre-calculated global protein sequence identity was > 20%. We ran each algorithm 10 times on each dataset and recorded the resulting stable set sizes and computation times in each case.

### Indygene

The Indygene front-end consists of a web application that was developed using the Web.Py framework [60]. The input to the Indygene back-end processing system is a job file, which contains the user-inputted gene list and other meta-information including the organism name, gene identifier type, timestamp and user details. If necessary, Affymetrix probe set data is used to convert microarray probeIDs to their corresponding gene names. The gene names are then compared with each other and with information from UniProtKB to ensure that each gene name/symbol is valid and unique.

Pre-computed protein similarity information for a selected set of organisms is used to construct an adjacency list representation of the gene graph. This data together with a user-selected %ID paralog cut-off is used to determine whether an edge exists between two genes in the graph. The greedy GMIN algorithm is then used to reduce the gene graph to a subset of vertices such that no edges remain. Finally, this solution is used to construct a stable, or sequence-independent, gene list file that can be downloaded by the user via the user interface.

### Statistical Significance of GSA Results Using Indygene

Alonso *et al. *[38] used the Affymetrix GeneChip platform to examine the gene expression patterns in *Arabidopsis *seedlings and apices, and determined 628 genes whose expression levels were significantly altered after treatment with exogenous ethylene. Similarly to these authors, we performed GSA on this dataset using Fisher's exact test to rank terms in the GO Biological Process ontology by their overrepresentation in these genes compared to the rest of the genes on the microarray. Apart from issues related to this method's assumption that genes are expressed independently, Alexa *et al. *[61] noted that the complex structure of the GO also introduces dependencies among GO terms in the DAG. At present there is no consensus on the most appropriate way to deal with this issue when performing GSA, so to circumvent it we restricted our analysis to Plant GO SLIM terms.

We then used Indygene to remove pair-wise paralogy relationships with protein sequence identity > 30% and repeated the above GSA. To quantify the differences between the two resulting ordered lists of GO terms i.e. before and after the reduction, we used a ranked correlation measure (Kendall's *τ*). Although researchers normally focus on the few statistically significant or highly ranked GO terms towards the top of the list, we considered the entire list so as to incorporate information about the change in relative ranking of all GO terms.

We determined the statistical significance of this difference by comparing the above correlation test statistic to the null distribution of correlation values resulting from all possible similar-sized gene reductions i.e. a randomisation test. Because the number of distinct gene reductions was prohibitively large we used Monte Carlo sampling, which considers a fixed number of randomly generated reductions instead of enumerating all possibilities. One thousand random 'samples' were used to generate an estimate of the correlation null distribution.

### Reanalysis of GSA Results Using Indygene

We reanalysed previously published gene expression datasets used by the authors of GoMiner, GSEA and SAM-GS to demonstrate the utility of their proposed GSA methods. We selected these three tools, each representing one of the three major categories of GSA methods discussed, to determine Indygene's usefulness across a broad range of methods. Using the original datasets we compared the GSA results obtained before and after removing paralogy relationships with protein sequence identity > 30% using Indygene.

Using GoMiner, we reanalysed the Common Variable Immune Deficiency (CVID) gene expression dataset published by Zeeberg *et al. *[62]. The authors used custom microarrays to measure the gene expression response to CD3 and CD28 antigens/antibodies in peripheral blood mononuclear cells (PBMC) from one CVID patient and six healthy donors. By comparisons to the healthy donor controls, they identified 57 genes that were significantly differentially expressed in the cells from the CVID patient. Using this information, we submitted the original and paralog-reduced gene lists for analysis using the High-Throughput GoMiner web interface. We also used GoMiner to reanalyse the human airway epithelial cell transcriptome dataset of Spira *et al. *[40]. The study involved the gene expression profiling of epithelial cell samples obtained at bronchoscopy from 85 subjects, 23 of which were healthy and had never smoked. The authors identified 2382 genes that were expressed in all of these healthy never-smokers. To find GO Biological Process terms enriched in these genes, we once again used the High-Throughput GoMiner web interface to submit the original and paralog-reduced gene lists for GSA.

Using the Java GSEA Desktop Application, we reanalysed the five different gene expression datasets covered in the article by Subramanian *et al. *[3]. The first dataset comprised mRNA expression profiles of lymphoblastoid cells from 15 males and 17 females, in which the authors aimed to identify cytogenetic gene sets (MSigDB:C1) and functional gene sets (MSigDB:C2) enriched in either gender. The second study involved the identification of targets of the transcription factor p53, which regulates the cell cycle in response to various cellular stress signals including DNA damage, thereby suppressing tumorogenesis. They used NCI-60 cancer cell lines to find functional gene sets (MSigDB:C2) enriched in the expression patterns of 17 classified as possessing the wild-type *p53 *gene when compared to that of 33 classified as carrying mutations in the gene [63], and vice-versa. Thirdly, they used GSEA and cytogenetic gene sets (MSigDB:C1) to find positions of frequent chromosomal alteration in acute lymphoid leukaemia (ALL) or acute myeloid leukaemia (AML). The dataset consisted of expression patterns obtained from 24 ALL patients and 24 AML patients [64]. Lastly, datasets from two independent studies were used to determine whether GSEA could identify functional gene sets (MSigDB:C2) correlated with clinical outcome in lung cancer. The Boston [65] and Michigan [66] studies measured gene expression levels in tumour samples from 62 and 86 patients with lung adenocarcinomas respectively, indicating patient survival outcome as either 'good' or 'poor'.

Using R code of the SAM-GS procedure made available by Dinu *et al. *[18], we reanalysed the 'p53 status' dataset of Subramanian *et al. *[3] using the original and paralog-reduced gene lists. Further details of this gene expression dataset are as indicated above.

## Authors' contributions

AJF carried out the research and drafted the manuscript. CS and NM conceived and directed the project and revised the manuscript. All authors read and approved the final manuscript.
